# Research on the Potential Mechanism of Guanine Nucleotides Enhancing the Tolerance of *Lactiplantibacillus plantarum* Y12

**DOI:** 10.3390/foods15122244

**Published:** 2026-06-22

**Authors:** Meichen Sui, Tianhao Zhang, Yaqi Hou, Xueqi Lu, Xiaochen Shi, Zhiyan Wen, Yanfeng Tuo, Guangqing Mu, Fang Qian, Yinglong Song, Xuemei Zhu

**Affiliations:** 1 School of Food Science and Technology, Dalian Polytechnic University, Dalian 116034, China; 2 State Key Laboratory of Food Science and Resources, Jiangnan University, 1800 Lihu Road, Wuxi 214122, China; 3 Dalian Probiotics Function Research Key Laboratory, Dalian Polytechnic University, Dalian 116034, China

**Keywords:** *Lactiplantibacillus plantarum*, biofilm formation, second messenger, fatty acid, transcriptomics

## Abstract

This study aims to elucidate the mechanism by which exogenous guanosine monophosphate (GMP) enhances the stress tolerance of *Lactiplantibacillus plantarum* Y12. Phenotypic assays demonstrated that GMP supplementation significantly improved biofilm formation, adhesion index, and auto-aggregation ability. The survival ability of Y12 in simulated gastric juice, intestinal juice, and freeze-drying stress was also significantly increased. Transcriptomic results revealed that GMP increased the intracellular content of the second messengers C-di-AMP and C-di-GMP by reducing phosphodiesterase (PDE, *RS04640*). This, together with the upregulated expression of *luxR* and *rpoN*, synergistically promoted biofilm formation. Furthermore, GMP enhanced acid tolerance by increasing glutamate decarboxylase activity (GAD, *RS05235*). It also significantly elevated the levels of extracellular proteins, exopolysaccharides, membrane polysaccharides, and membrane fatty acids by modulating genes related to proteins (*yidC*, *yajC*), polysaccharides (*agaB*, *agaC*), and membrane fatty acid synthesis (*RS02005*, *plsY*), which was also demonstrated by quantitative determination. Collectively, these regulatory mechanisms substantially improve the stress tolerance of *L. plantarum* Y12, providing a theoretical basis for its application.

## 1. Introduction

Probiotics are a type of live microorganisms that can bring benefits to the health of their hosts. Although the global probiotic industry has developed rapidly in recent years, there are still practical problems in the process from probiotic production and processing to actual application. These problems are caused by the insufficient ability of probiotic strains to cope with various environmental stress factors, resulting in unstable functional activity. From production to application, the accumulation of metabolites during large-scale cultivation of probiotics significantly restricts their own growth; then, during the production of probiotic powder through drying technology, stress from various processing-related environmental factors greatly reduces the survival rate of probiotics [1]. In addition, the environmental factors in the host’s stomach (low pH, pepsin) and small intestine (bile acids, digestive enzymes) also cause a large number of probiotics to become inactive. The probiotics still need to competitively adhere and colonize with the intestinal flora [2,3]. Therefore, improving the survival ability of probiotics under various adverse environmental stress factors can help solve the application and development bottlenecks of probiotics.

The stress tolerance of probiotics is closely linked to their growth-related characteristics, including cell wall synthesis, the production of bioactive macromolecules (exopolysaccharides, proteins, and fatty acids), and biofilm formation [4]. The extracellular polymeric matrix of biofilms serves as a protective barrier and enhances adhesion [5,6], thereby supporting resistance to environmental stressors. In many organisms, nucleotides function as intracellular signaling molecules that mediate environmental responses and regulate metabolism [7]. The identified second messenger molecules such as cyclic di-adenosine monophosphate and cyclic di-guanosine monophosphate are known to modulate bacterial stress responses and biofilm formation [8]. Current evidence indicates that nucleotide signaling pathways exert their effects by interacting with proteins or RNA, thereby influencing transcription, translation, and enzyme activity [9,10], and ultimately driving changes in physiology, metabolism, and growth phenotype.

The probiotic strain *Lactiplantibacillus plantarum* Y12, independently isolated by the research group, could respond to polysaccharides, including self-synthesized exopolysaccharides and various food-derived polysaccharides, as signal-like molecules to regulate growth and metabolism, thereby enhancing stress resistance. This is evidenced by significantly improved survival under freeze-drying and gastrointestinal digestion. Integrated transcriptomic and metabolomic analyses revealed that polysaccharides promote the accumulation of the intracellular second messenger cyclic diguanylate, which activates quorum sensing and drives the high-level synthesis of bioactive macromolecules such as extracellular polysaccharides, teichoic acids, and long-chain membrane fatty acids. These findings suggest a correlation between nucleotide metabolism and stress resistance in *L. plantarum* Y12. In this study, Y12 was used as the model strain, and exogenous guanosine monophosphate (GMP) was applied to modulate intracellular nucleotide metabolism. By examining phenotypic traits, fatty acid synthesis, and gene expression, the key role of nucleotide metabolism in stress resistance was elucidated. Clarifying this regulatory mechanism may help address current bottlenecks in the application and development of probiotic strains.

## 2. Materials and Methods

### 2.1. Strain and Cell Culture Activation

*Lactiplantibacillus plantarum* Y12 was obtained from the Dalian Key Laboratory of Probiotic Functional Characteristics. For strain activation, the frozen stock was inoculated at a volume ratio of 2% (*v*/*v*), which was achieved by transferring 100 μL of the bacterial suspension into 5 mL of fresh sterile MRS broth (Basebio Biotechnology Co., Ltd., Hangzhou, China). The culture was incubated at 37 °C for 16 h, and this subculture procedure was repeated three times consecutively to obtain fully activated bacterial cells for subsequent experiments.

MRS broth composition: peptone 10 g/L, beef extract powder 8 g/L, yeast extract powder 4 g/L, glucose 20 g/L, dipotassium hydrogen phosphate 2 g/L, sodium acetate 5 g/L, diammonium hydrogen citrate 2 g/L, magnesium sulfate 0.2 g/L, manganese sulfate 0.04 g/L, Tween 80 1.0 mL/L, pH 5.7 ± 0.2.

The cells used in the experiment were human colon cancer HT-29 cells, purchased from the Shanghai Cell Bank of the Chinese Academy of Sciences. Cells were cultured in RPMI-1640 (Grand Island Biological Company, New York, NY, USA) medium supplemented with 10% heat-inactivated fetal bovine serum (56 °C, 30 min) and 1% penicillin–streptomycin, and maintained at 37 °C in a humidified 5% CO_2_ incubator.

### 2.2. Quantification of Biofilm Formation

Biofilm formation was quantified using the crystal violet microtiter plate assay. *L. plantarum* Y12 was inoculated (2%, *v*/*v*) into MRS broth supplemented with varying concentrations of guanosine monophosphate (GMP; 25, 50, 100, and 200 μM). MRS broth without GMP served as the control. The 96-well plates were incubated statically at 37 °C for 16 h. After incubation, the medium was discarded, and the wells were gently washed three times with sterile phosphate-buffered saline (0.1 M, pH 7.3). The plates were air-dried, and the adherent biofilms were fixed with 200 μL of 95% methanol for 15 min. After removing the fixative and air-drying, the biofilms were stained with 200 μL of 2% (*w*/*v*) crystal violet for 5 min. Excess staining was removed by rinsing with deionized water. The bound dye was then solubilized with 160 μL of 33% (*v*/*v*) glacial acetic acid. A 125-μL aliquot was transferred to a new plate, and the absorbance was measured at 590 nm [11]. Biofilm formation was calculated using the following formula.

Biofilm formation ratio (%) = (ODexperimental/ODcontrol) × 100

### 2.3. Growth Curve Analysis

The effect of GMP on bacterial growth was assessed using an automatic growth curve analysis system. *L. plantarum* Y12 was inoculated (2%, *v*/*v*) into MRS broth supplemented with varying concentrations of GMP (25, 50, 100, and 200 µM). Growth was monitored continuously at 37 °C for 24 h, with the optical density (OD_600_) recorded automatically at 1 h intervals.

### 2.4. Determination of Survival Ability in Simulated Gastrointestinal Fluids

Simulated gastric fluid (SGF) was prepared by dissolving pepsin in 0.1 mol/L PBS (pH 7.3) at a final concentration of 3.0 g/L, adjusting the pH to 2.5, and sterilizing by filtration through a 0.22 μm sterile membrane. Simulated intestinal fluid (SIF) was prepared by dissolving trypsin in 0.1 mol/L PBS (pH 7.3) at a final concentration of 1.0 g/L, supplementing with 1.8% (*w*/*v*) bovine bile salts (Solarbio Science & Technology Co., Ltd., Beijing, China), and sterilizing by filtration through a 0.22 μm sterile membrane. Activated *L. plantarum* Y12 was inoculated (2%, *v*/*v*) into MRS broth with or without 200 µM GMP supplementation and incubated at 37 °C for 16 h. The bacterial cells were harvested by centrifugation (4000× *g*, 4 °C, 10 min), washed three times with sterile 0.1 mol/L PBS (pH 7.3), and resuspended in an equal volume of sterile PBS to obtain the original bacterial suspension. An aliquot (500 μL) of the original suspension was added to 4.5 mL of SGF and incubated at 37 °C in a water bath for 3 h. Subsequently, 500 μL of the SGF-treated culture was transferred to 4.5 mL of SIF and incubated at 37 °C in a water bath for an additional 8 h. Viable cell counts (CFU/mL) in the original suspension and after sequential SGF and SIF digestion were determined using the pour plate method. The survival rate was calculated according to the following formula.

Survival rate (%) = (N_1_/N_0_) × 100

N_0_ initial viable cell count (CFU/mL)

N_1_ viable cell count after gastric or gastrointestinal digestion (CFU/mL)

### 2.5. Determination of Freeze-Drying Survival Ability

Activated *L. plantarum* Y12, cultured in MRS broth with or without 200 µM GMP for 16 h, was harvested by centrifugation (4000× *g*, 4 °C, 10 min), washed three times with sterile PBS (0.1 M, pH 7.3), and resuspended in an equal volume of the same buffer. The suspension was pre-frozen at −40 °C for 2 h and then freeze-dried at −60 °C for 72 h. The lyophilized powder was rehydrated with an equal volume of physiological saline (relative to the pre-centrifugation volume). Viable cell counts were determined by the pour plate method. Only plates with 30–300 colonies were counted. Colony-forming unit (CFU) values are means of three replicates. The freeze-drying survival rate was calculated as [12]:

Freeze-drying survival rate (%) = (A_1_/A_2_) × 100

A_1_—viable cell counts after freeze-drying (CFU/mL);

A_2_—viable cell counts before freeze-drying (CFU/mL).

### 2.6. Determination of Adhesion Ability to HT-29 Cells

The adhesion ability of GMP-treated *L. plantarum* Y12 to HT-29 cells was examined using an in vitro intestinal epithelial model. HT-29 cells were seeded into 12-well plates at 5 × 10^5^ cells per well and cultured at 37 °C in 5% CO_2_ until 80–90% confluence. Activated *L. plantarum* Y12, cultured in MRS broth with or without 200 µM GMP for 16 h, was harvested by centrifugation (4000× *g*, 4 °C, 10 min), washed three times with sterile PBS (0.1 M, pH 7.3), and resuspended in RPMI-1640 medium at 2 × 10^9^ CFU/mL. HT-29 monolayers were washed three times with sterile PBS. Then, 900 μL of RPMI-1640 and 100 μL of the bacterial suspension were added to each well and incubated at 37 °C in 5% CO_2_ for 2 h. After incubation, the wells were gently washed three times with PBS to remove non-adherent bacteria. Adhered bacteria were released by adding 1 mL of 0.5% Triton X-100, incubating on ice for 10 min, and pipetting vigorously to lyse the cells. The lysate was collected, serially diluted (10-fold), and plated onto MRS agar. After 48 h of incubation at 37 °C, colonies were counted to determine the number of adherent bacteria. The adhesion index was calculated as follows [13]:

Adhesion index = (Number of adherent *L. plantarum* Y12 CFU)/(Number of HT-29 cells per well)

### 2.7. Determination of Auto-Aggregation Ability

Activated *L. plantarum* Y12 was inoculated (2%, *v*/*v*) into MRS broth with or without 200 µM GMP supplementation and incubated at 37 °C for 16 h. The bacterial cells were harvested, washed, and resuspended in sterile PBS, and the optical density was adjusted to 1.00 ± 0.02 at 600 nm (A_0_). The suspension was allowed to stand at room temperature, and the absorbance of the upper layer (A_t_) was measured after 30 min of incubation. The auto-aggregation percentage was calculated using the following formula [14].

Auto-aggregation (%) = [(A_0_ − A_t_)/A_0_] × 100

### 2.8. Determination of Intracellular Second Messenger Content

*L. plantarum* Y12, cultured in MRS broth with or without 200 µM GMP for 16 h, was harvested by centrifugation (4000× *g*, 4 °C, 10 min). The cell pellet was washed three times with ice-cold 1 mM ammonium acetate and resuspended in an equal volume of ice-cold acetonitrile–methanol–water (40:40:20, *v*/*v*/*v*). The suspension was subjected to ultrasonic lysis in an ice-water bath for 2 h (37 kHz, 100% power). Cell debris was removed by centrifugation (10,000× *g*, 4 °C, 10 min), and the supernatant containing cyclic dinucleotides was collected. The pellet was re-extracted three times with the same solvent [15]. The supernatants were pooled, concentrated by rotary evaporation, and reconstituted with deionized water. Concentrations of C-di-GMP and C-di-AMP were determined using commercial microbial cyclic dinucleotide assay kits (Ruichuang Biotechnology Co., Ltd., Tianjin, China).

### 2.9. Extraction of Bacterial Metabolites

*L. plantarum* Y12 suspension was inoculated (2%, *v*/*v*) into MRS broth with or without 200 µM GMP supplementation and incubated statically at 37 °C for 16 h. The culture was centrifuged (10,000× *g*, 4 °C, 10 min) to separate the supernatant and cell pellet. The cell pellet was washed once with sterile 0.1 M PBS (pH 7.3) and resuspended in 0.9% NaCl solution. The bacterial suspension was incubated in a 60 °C water bath for 30 min. After centrifugation (10,000× *g*, 4 °C, 10 min), the resulting supernatant was collected as the extracted cell surface polymer matrix solution.

### 2.10. Determination of Protein and Polysaccharide Contents

The protein and polysaccharide contents in both the culture supernatant and the extracted cell surface polymer matrix solution were determined. Protein concentration was measured using a BCA assay kit (Solarbio Science & Technology Co., Ltd., Beijing, China). Polysaccharide content was quantified using the phenol–sulfuric acid method [16].

### 2.11. Determination of Cell Membrane Fatty Acid Content and Composition

The cell membrane polymer matrix solution (500 μL) was transferred to 1 mL of dichloromethane: methanol (1:1, *v*/*v*), subjected to low-temperature ultrasonication for 15 min, and then incubated at −20 °C for 15 min. After centrifugation (13,000× *g*, 4 °C, 10 min), the lower layer was collected into a 1.5 mL EP tube and dried using nitrogen gas. The residue was mixed with 0.5 mL of methylating reagent (0.5 M NaOH in methanol), vortexed for 30 s, and incubated at 60 °C for 30 min. After cooling, 0.5 mL of n-hexane was added, followed by vortexing for 30 s and centrifugation (13,000× *g*, 4 °C, 10 min). A 200 μL aliquot of the upper (n-hexane) layer was transferred to an injection vial for GC-MS (Agilent TechnoloGies, Inc., Santa Clara, CA, USA) analysis. GC-MS analysis was performed using an Agilent DB-FastFAME capillary column (20 m × 0.18 mm × 0.2 μm). High-purity helium (≥99.999%) was used as the carrier gas at a flow rate of 1.0 mL/min. The injector temperature was 230 °C, and the injection volume was 1 μL with a split ratio of 50:1 (solvent delay: 1.0 min). The oven temperature program was as follows: initial hold at 80 °C for 0.5 min, increased to 175 °C at 70 °C/min, then to 230 °C at 8 °C/min, and held for 1 min. The mass spectrometer was operated in electron impact (EI) mode at 70 eV, with the ion source, quadrupole, and transfer line temperatures set at 230 °C, 150 °C, and 240 °C, respectively. Data were acquired in selected ion monitoring (SIM) mode [17].

### 2.12. Transcriptomics Analysis

Total RNA was extracted from *L. plantarum* Y12 cultured in MRS broth with or without 200 μM GMP at 37 °C for 16 h using TRIzol reagent (Invitrogen, Carlsbad, CA, USA). RNA concentration, purity, and integrity were assessed using a NanoDrop spectrophotometer (Thermo Fisher Scientific, Waltham, MA, USA) and an Agilent 2100 Bioanalyzer (Agilent Technologies, Santa Clara, CA, USA). Ribosomal RNA was depleted using the Zymo-Seq RiboFree Total RNA Library Kit (Zymo Research, Irvine, CA, USA), and the enriched mRNA was fragmented using divalent cations. First-strand cDNA was synthesized with random oligonucleotides and SuperScript III reverse transcriptase (Invitrogen), followed by second-strand synthesis using DNA Polymerase I and RNase H (Invitrogen). Double-stranded cDNA was purified with AMPure XP beads (Beckman Coulter, Brea, CA, USA), end-repaired, 3′-adenylated, and ligated with Illumina sequencing adapters. cDNA fragments of 400–500 bp were size-selected by AMPure XP beads, amplified with Illumina PCR Primer Cocktail for 15 cycles, and purified again. Library quality was verified on an Agilent 2100 Bioanalyzer, and quantification was performed using the Quant-iT PicoGreen dsDNA Kit (Thermo Fisher Scientific) on a Quantifluor-ST fluorometer (Promega, Madison, WI, USA) and qPCR on a StepOnePlus Real-Time PCR System (Applied Biosystems, Foster City, CA, USA). Qualified libraries were pooled and sequenced on an Illumina NovaSeq 6000 platform (Illumina, San Diego, CA, USA) in PE150 mode [18,19].

Raw reads were filtered using fastp (v0.22.0) and aligned to the *L. plantarum* reference genome via Bowtie2 (v2.5.1). Gene expression levels were quantified, and relative mRNA abundance was calculated as FPKM using HTSeq (v0.9.1). Differential expression analysis between the two groups of samples was performed using the DESeq2 R package (v1.38.3). DESeq2 provides statistical methods for identifying differential expression in digital gene expression data based on a negative binomial distribution model. The resulting *p*-values were then adjusted using the Benjamini and Hochberg method to control the false discovery rate. Genes with an adjusted *p*-value < 0.05 identified by DESeq2 were defined as differentially expressed genes (DEGs) [20]. Principal component analysis (PCA) was performed based on the expression matrix of DEGs, and the calculation and visualization were conducted in R using the built-in plotPCA function of the DESeq2 package [21]. Hierarchical clustering analysis was performed using the ComplexHeatmap package (v2.16.0). Volcano plot analysis was performed using the ggplot2 package (v3.4.4). GO and KEGG enrichment analyses were carried out using clusterProfiler (v4.6.0), and the enrichment results were visualized using the Circlize package (v0.4.15). Raw sequencing data were deposited in the NCBI Sequence Read Archive (SRA) under BioProject submission number PRJNA1459008.

### 2.13. Statistical Analysis

All experiments were repeated three times. The experimental data were analyzed using IBM SPSS Statistics version 20.0.0 software. The significance of data differences was assessed using one-way analysis of variance (ANOVA), with *p* < 0.05. All data results are expressed as mean ± standard deviation.

## 3. Results and Discussion

### 3.1. The Effect of GMP on the Biofilm Formation and Growth of Y12

**Figure 1:**
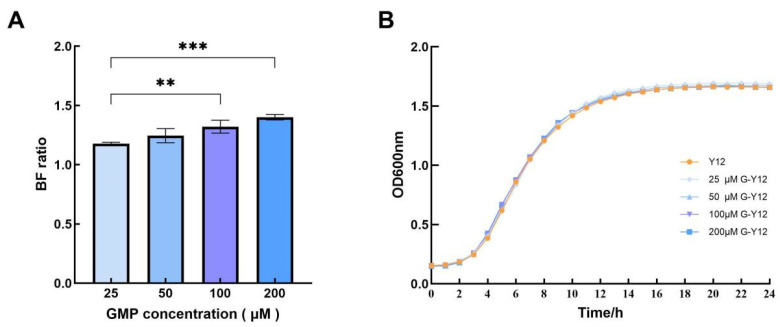
Effects of different concentrations of GMP on biofilm formation (**A**) and growth curve (**B**) of *L. plantarum* Y12. Note: “**”, “***” on bars indicate significant differences, *p* < 0.01, *p* < 0.001, respectively.

Cyclic di-GMP (c-di-GMP) is an essential second messenger in bacteria, playing a crucial role in the regulation of bacterial behavior and environmental adaptation [22,23]. As a hydrolysis product of C-di-GMP, GMP has a certain impact on the accumulation of C-di-GMP [24,25]. Therefore, the biofilm formation and growth of Y12 were investigated after the exogenous addition of GMP. In the results shown in Figure 1A, GMP promoted biofilm formation of *L. plantarum* Y12 in a dose-dependent manner. The Y12 supplemented with 200 μM GMP exhibited the highest biofilm formation ratio (1.402) (*p* < 0.001). In the growth curve shown in Figure 1B, *L. plantarum* Y12 entered the logarithmic phase after 4 h and reached the stationary phase at 12 h. The bacterial strain showed a rapid linear rise in OD_600_ after 4 h of incubation, indicating the onset of the rapid growth phase. GMP addition did not significantly affect growth, indicating that the increase in biofilm formation was not due to an increased cell number, but rather resulted from GMP-mediated molecular regulation.

### 3.2. The Effect of GMP on the Cell Adhesion and Auto-Aggregation Abilities of Y12

**Figure 2:**
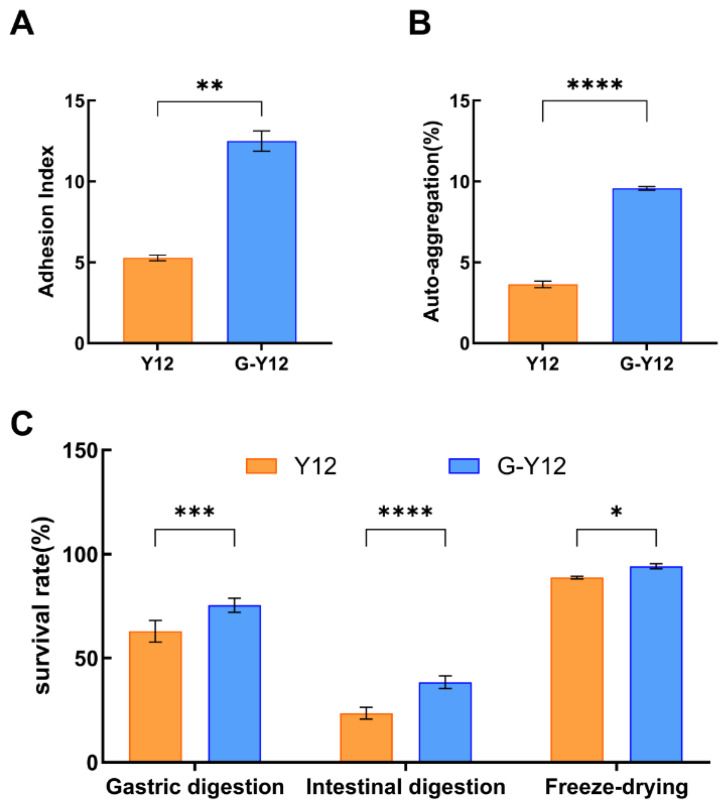
Effects of GMP on adhesion (**A**), auto-aggregation (**B**), and stress tolerance (**C**) of *L. plantarum* Y12. Note: “*”, “**”, “***”, “****” on bars indicate significant differences, *p* < 0.05, *p* < 0.01, *p* < 0.001, *p* < 0.0001, respectively.

Adhesion and colonization are critical for probiotic function. To assess the effect of GMP on the adhesion of *L. plantarum* Y12, HT-29 cells were used as an in vitro model. As shown in Figure 2A, the adhesion index of the G-Y12 group was significantly higher than that of the Y12 group (*p* < 0.01), increasing from 5.47 to 12.50. This increase may be attributed to GMP-induced changes in surface-active substances, including lipoteichoic acid, adhesion proteins, polysaccharides, glycolipids, and glycoproteins. Additionally, GMP significantly enhanced the auto-aggregation ability of *L. plantarum* Y12 (Figure 2B) (*p* < 0.0001).

### 3.3. The Effect of GMP on the Stress Resistance of Y12

The survival rate of *L. plantarum* Y12 after freeze-drying and simulated gastrointestinal digestion was evaluated. As shown in Figure 2C, with exposure to simulated gastric juice, the survival rate of G-Y12 (75.39%) was significantly higher than that of Y12 (62.85%) (*p* < 0.05). Similarly, after simulated intestinal juice digestion, the survival rate of G-Y12 (38.38%) remained significantly elevated (*p* < 0.01). These results suggest that GMP treatment enhances the gastrointestinal tolerance of *L. plantarum* Y12, increasing the likelihood that sufficient viable bacteria reach the intestine to colonize and exert probiotic effects. Additionally, under freeze-drying stress, the survival rate of the G-Y12 group significantly increased to 94.11%%, compared to 88.69% in the Y12 group (*p* < 0.0001). This enhanced tolerance suggests improved stability of G-Y12, rendering it more suitable for processing and application.

### 3.4. The Effect of GMP on the Second Messenger Content of Y12

**Figure 3:**
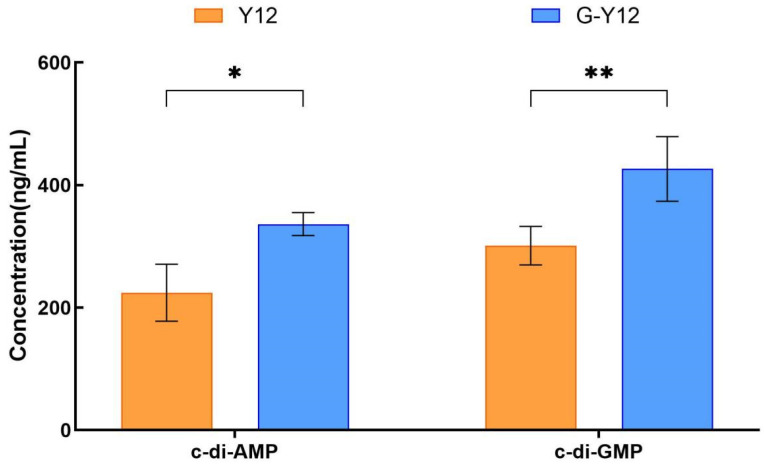
Effect of GMP on the content of the second messenger in *L. plantarum* Y12. Note: “*”, “**”, *p* < 0.05, *p* < 0.01, respectively.

C-di-AMP and C-di-GMP are key second messengers involved in regulating bacterial motility and biofilm formation [26,27]. As shown in Figure 3, the intracellular concentrations of both molecules were significantly higher in G-Y12 than in the original strain Y12. Specifically, c-di-AMP levels increased from 224.53 ng/mL in Y12 to 336.39 ng/mL in G-Y12 (*p* < 0.05), and c-di-GMP levels rose from 301.39 ng/mL to 426.52 ng/mL (*p* < 0.01). The increase in the content of both second messengers contributes to the biofilm formation of Y12, and is also related to the ability of Y12 to withstand environmental stress factors such as freeze-drying and simulated gastrointestinal fluid digestion.

### 3.5. The Effect of GMP on the Protein and Polysaccharide Content of Y12

**Figure 4:**
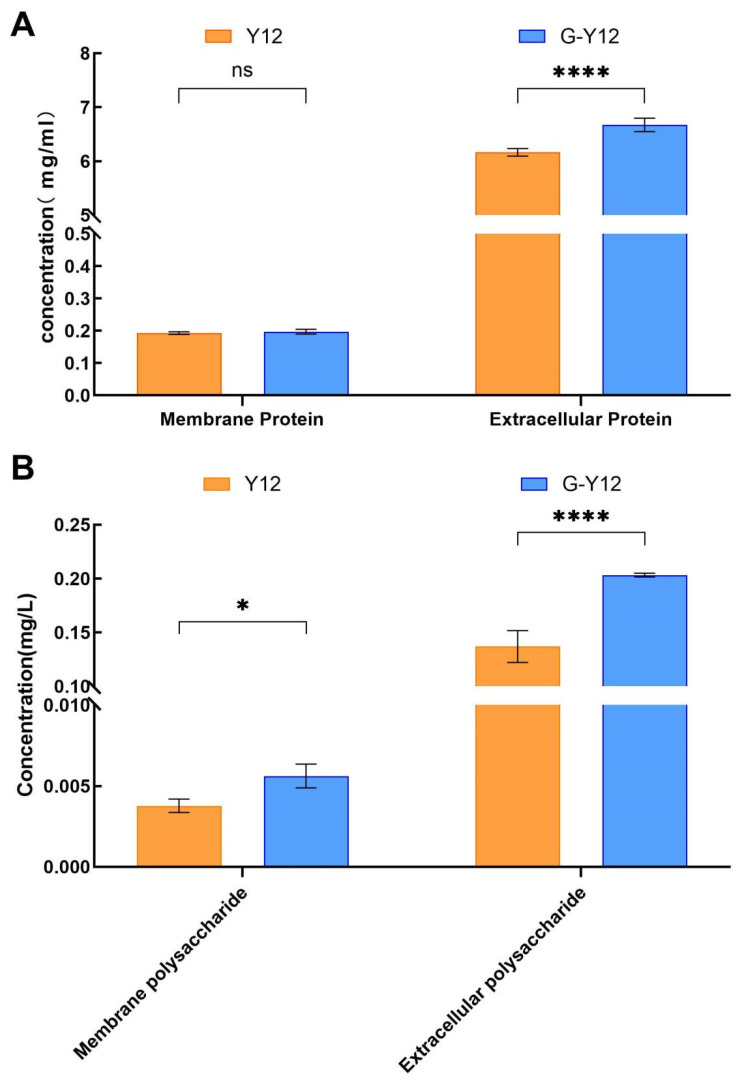
Effects of GMP on membrane protein (**A**) and polysaccharide (**B**) contents in *L. plantarum* Y12. Note: ns on bars indicate no significant differences; “*”, “****” on bars indicate significant differences, *p* < 0.05, *p* < 0.0001, respectively.

Furthermore, the synthesis levels of the biological macromolecular substances were investigated. In the results shown in Figure 4A, membrane protein levels were comparable between G-Y12 and Y12, but extracellular protein concentration was significantly higher in G-Y12 (6.67 mg/mL) than in Y12 (6.16 mg/mL; *p* < 0.0001). In addition, both membrane and extracellular polysaccharide concentrations were significantly increased in G-Y12 (Figure 4B). Membrane polysaccharide levels reached 0.00562 mg/L in G-Y12 versus 0.00378 mg/L in Y12 (*p* < 0.05), and extracellular polysaccharide levels increased from 0.137 mg/L in Y12 to 0.203 mg/L in G-Y12 (*p* < 0.0001). Extracellular proteins and polysaccharides are key components of the biofilm matrix, contributing to its structure and stability. The significant increase in these components in G-Y12 is consistent with its enhanced biofilm formation (Figure 1A). Elevated C-di-GMP levels in G-Y12 (Figure 3) are known to promote extracellular matrix synthesis, suggesting that C-di-GMP upregulation may drive the increase in extracellular proteins and polysaccharides, thereby supporting the formation of a more stable biofilm. Membrane polysaccharides contribute to cell membrane integrity and function, which may enhance the stress tolerance observed in G-Y12 (Figure 2C). The increase in membrane polysaccharides in G-Y12 likely provides additional protection against environmental stresses, such as gastrointestinal digestion and freeze-drying.

### 3.6. The Effect of GMP on the Fatty Acid Composition of the Cell Membrane of Y12

Membrane fatty acids play a significant role in the stress resistance of bacteria [28,29]. Compared with the Y12 group, the membrane fatty acid profile of the G-Y12 group showed significantly different changes (Table 1). A total of 27 types of fatty acids were detected, and 23 types were upregulated in G-Y12, while only four (myristoleate, 11-14 eicosadienoate, caprate and caprylate) were downregulated. Notably, palmitate (C_16_H_32_O_2_) and linoelaidate (C_18_H_32_O_2_) were significantly increased (*p* < 0.01). In addition, oleate (C_18_H_34_O_2_), vaccenate (C_18_H_34_O_2_), myristate (C_14_H_28_O_2_), 10-transnonadecenoate (C_19_H_36_O_2_), linoleate (C_18_H_32_O_2_), palmitelaidate (C_16_H_30_O_2_), petroselaidate (C_18_H_34_O_2_), and elaidate (C_18_H_34_O_2_) were also significantly upregulated (*p* < 0.05). In G-Y12, the significant increase in unsaturated fatty acids (oleate, linoleate), and saturated fatty acid (palmitate), are particularly notable.

### 3.7. GMP’s Effect on the Stress Resistance of Y12 Based on Transcriptomics Analysis

**Figure 5:**
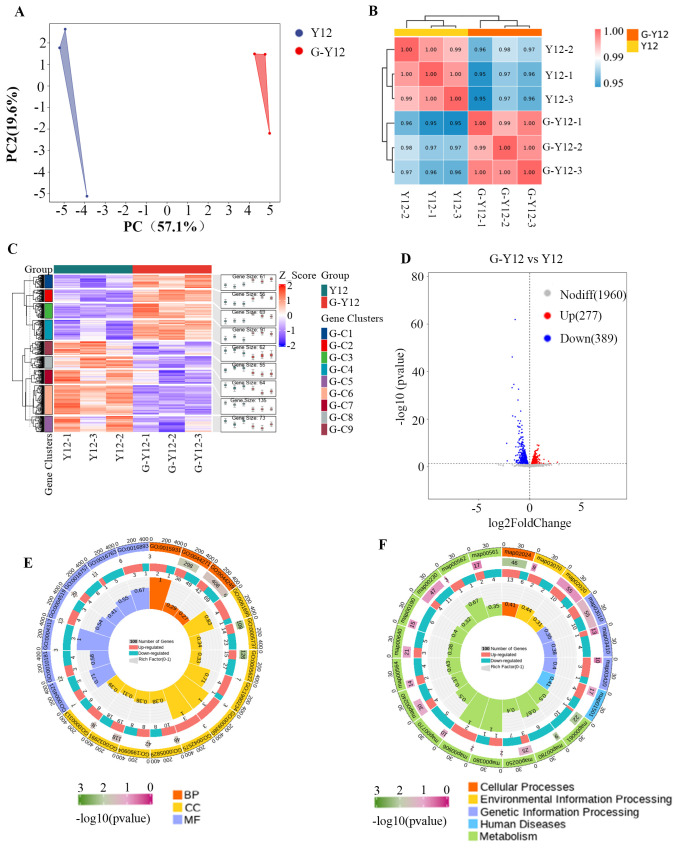
Effect of GMP on the transcriptomic profile of *L. plantarum* Y12. (**A**) PCA, (**B**) Pearson correlation analysis, (**C**) heatmap of differentially expressed genes, (**D**) volcano plot of differentially expressed genes, (**E**) GO enrichment analysis, (**F**) KEGG enrichment analysis. Note: The circles are viewed from the outside inward. The first circle shows pathway levels (different colors for different levels) with an external coordinate scale for gene counts. The second circle displays background gene counts annotated to each pathway and enrichment *p*-values: longer bars mean more genes, and smaller *p*-values correspond to redder colors. The third circle is a bar chart of upregulated/downregulated genes (red = upregulated, blue = downregulated) with specific values below; if only one column of differentially expressed genes is input (no up/down distinction), it shows the total count. The fourth circle presents the Rich Factor (differentially expressed genes enriched in the pathway/total annotated genes per pathway), with each small grid of the background auxiliary line indicating 0.1.

To investigate the molecular basis of phenotypic differences between G-Y12 and Y12, transcriptome analysis was performed. PCA revealed a clear separation between two groups, with PC1 and PC2 accounting for 57.1% and 19.6% of the total variance, respectively (Figure 5A). High intra-group Pearson correlation coefficients (>0.95) confirmed the reproducibility of the transcriptome data (Figure 5B). Hierarchical clustering analysis revealed distinct expression profiles between Y12 and G-Y12 (Figure 5C). Volcano plot analysis identified 666 differentially expressed genes (DEGs) (*p* < 0.05), of which 277 were upregulated and 389 were downregulated in G-Y12 (Figure 5D).

GO enrichment analysis was performed on the differentially expressed genes. As shown in Figure 5E, DEGs were significantly enriched in the transferase complex involved in phosphorus-containing group transfer (GO:0061695) and the cytoplasm (GO:0005737), suggesting that the gene products are primarily cytoplasmic and reflect intracellular metabolic reprogramming in response to stress. In the molecular function category, DEGs were enriched in endoribonuclease activity (GO:0004521) and FMN binding (GO:0010181), indicating changes in genes related to nucleic acid hydrolysis. In the biological process category, DEGs were significantly enriched in nucleobase-containing compound transport (GO:0015931) and cellular nitrogen compound biosynthetic process (GO:0044271), implicating these genes in core metabolic and biosynthetic pathways.

KEGG enrichment analysis revealed that DEGs were significantly enriched in quorum sensing (map02024), fatty acid synthesis (map00061), and biotin metabolism (map00780). Additional enriched pathways included bacterial secretion systems (map03070), two-component systems (map02020), and glycerophospholipid metabolism (map00564), consistent with the observed increases in biofilm formation, stress tolerance, and membrane fatty acids (Figure 1A and Figure 2C, Table 1). Pathways related to nucleotide metabolism–pyrimidine (map00240) and purine metabolism (map00230), as well as oxidative phosphorylation (map00190) were also differentially enriched (Figure 5F).

**Figure 6:**
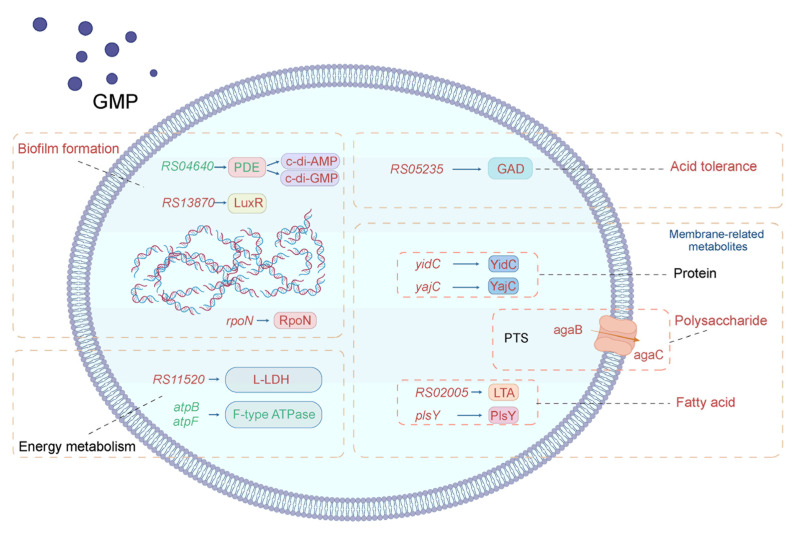
Regulatory mechanism initiated by guanine addition in Y12. Note: Red text represents increased expression and green text represents decreased expression. Key pathways and components involved in the protective response include biofilm formation-related factors (LuxR family transcriptional regulator RS13870, cyclic nucleotide phosphodiesterase PDE/RS04640, RNA polymerase sigma factor RpoN/RS09475), acid tolerance-related glutamate decarboxylase GAD/RS05235, energy metabolism-related enzymes (L-lactate dehydrogenase L-LDH/RS11520, F-type ATPase subunits atpB and atpF), and membrane-related metabolite synthesis-related proteins (membrane protein insertase YidC, preprotein translocase subunit YajC, phosphotransferase system (PTS) components agaB and agaC, glycerol-3-phosphate acyltransferase PlsY, and LTA synthase family gene *RS02005*).

The key differentially expressed genes (DEGs) that saw significant changes in Y12 after the exogenous addition of GMP are shown in Table 2. Significantly upregulated DEGs include *RS13870* (LuxR family transcriptional regulator), *yidC* (membrane protein insertase YidC), *yajC* (preprotein translocase subunit YajC), *RS05235* (glutamate decarboxylase), *dltA* (D-alanine--poly(phosphoribitol) ligase subunit DltA), *rpoN* (RNA polymerase factor sigma-54), *murB* (UDP-N-acetylmuramate dehydrogenase), *agaB* (PTS galactosamine transporter subunit IIB), *agaC* (PTS galactosamine transporter subunit IIC), *plsY* (1-O-acyltransferase PlsY), *RS02005* (LTA synthase family protein), and *RS11520* (L-lactate dehydrogenase). Significantly downregulated DEGs include *RS04640* (3′,5′-cyclic-nucleotide phosphodiesterase), *atpB* (F0F1 ATP synthase subunit A), and *atpF* (F0F1 ATP synthase subunit B). Therefore, the addition of GMP has a significant impact on the growth characteristics and related metabolic characteristics of Y12. A summary of the main findings is presented in Figure 6; exogenous supplementation of GMP promoted the accumulation of C-di-AMP and C-di-GMP, thereby facilitating the biofilm formation. Meanwhile, GMP enhanced the energy metabolism; the synthesis ability of proteins, polysaccharides, and fatty acids; and the acid tolerance of Y12.

## 4. Discussion

As is well known, the functional activity of probiotics is closely related to their survival and colonization capabilities [30]. Biofilms, as a form of bacterial growth pattern, exhibit a natural ability to protect themselves against external environmental stress [31]. Therefore, the application of biofilm-based probiotics has gained increasing attention [32]. The second messenger is a versatile signaling molecule widely present in bacteria. It can combine with other complete metabolic pathways (such as phosphorylation and quorum sensing) to regulate the life processes of bacteria [33]. Cyclic-di-AMP and Cyclic-di-GMP are two types of second messengers that are widely present in bacterial cells [34].

The Cyclic-di-GMP monomer exhibits a double symmetry, with the two GMP parts linked by a 5′–3′ large ring. The dual guanosine diphosphate cyclase (DGC) catalyzes the synthesis of Cyclic-di-GMP through the cooperative action of its two catalytic GGDEF domains. Phosphodiesterases (PDEs) with EAL or HD-GYP domains can respectively hydrolyze Cyclic-di-GMP into 5′-phosphoguanosine-(3′-5′)-guanosine (pGpG) or GMP. Cyclic-di-GMP regulates various cellular processes by binding to effector molecules, including motility, adhesion, and biofilm formation [35].

Similarly, C-di-AMP is synthesized from two ATP molecules through the adenosine diphosphate cyclase (DAC). Although C-di-AMP is crucial for the growth and metabolism of bacteria, its excessive accumulation can still impair the function of the cells. Specific phosphodiesterase (PDE) can sense the increased concentration of C-di-AMP and degrade it into linear adenosine 5′-pApA or AMP molecules [36]. C-di-AMP also has a wide range of regulatory functions, which are used to control osmotic pressure, balance central metabolism, and influence the formation of biological membranes [37].

In this study, the purpose of the exogenous addition of GMP is to influence the synthesis or decomposition of intracellular second messengers, thereby interfering with a series of life activities of Y12, including biofilm formation and the synthesis of various bioactive substances. These changes will prompt alterations in Y12′s ability to cope with environmental stressors and its capacity for adhesion and colonization. Thus, the results showed that increased C-di-AMP concentration in G-Y12 may contribute to its enhanced stress tolerance (Figure 2C). Elevated C-di-GMP levels are closely associated with biofilm formation and extracellular matrix synthesis, consistent with the observed increase in biofilm formation by G-Y12 (Figure 1A). Thus, the upregulation of these second messengers provides a molecular basis for the improved biofilm formation and stress tolerance in G-Y12.

Notably, the synthesis level of polysaccharides secreted by Y12 was significantly increased under the influence of GMP, both in the supernatant and in the polymeric matrix. This is inseparably linked to the improvement in probiotics’ tolerance for gastrointestinal fluids and their ability to undergo freeze-drying [38,39]. Furthermore, membrane fatty acid composition is a key determinant of membrane fluidity, integrity, and function, and its modulation serves as an important adaptive response to environmental stress [40]. In G-Y12, the significant increase in unsaturated fatty acids (oleate, linoleate), and saturated fatty acid (palmitelaidate), are particularly notable. Unsaturated fatty acids enhance membrane fluidity via their double bonds, which has been shown to improve resistance to stresses including acid, osmotic pressure, and freeze-drying [41]. This finding is consistent with the enhanced stress tolerance observed in G-Y12 (Figure 2C).

Transcriptomics also supported the above-mentioned phenotypic phenomena based on the determination results of the gene expression levels of Y12 under GMP influence. Intracellular levels of cyclic dinucleotides are regulated by diguanylate cyclase (DGC) and phosphodiesterase (PDE). In G-Y12, the gene RS04640, encoding a 3′,5′-cyclic nucleotide phosphodiesterase, was downregulated, reducing the degradation of C-di-AMP and C-di-GMP and leading to their accumulation (Figure 3). This increase, together with the upregulation of biofilm-related genes such as *luxR* [27,42] and *rpoN* [43], likely promotes biofilm formation and enhances stress resistance in *L. plantarum* Y12.

The upregulation of glutamate decarboxylase (RS05235) in G-Y12 contributes to its enhanced acid tolerance. This enzyme converts glutamate to γ-aminobutyric acid (GABA), helping to neutralize intracellular acidity and maintain pH balance, thereby increasing survival under acidic conditions such as those in simulated gastric juice [44].

GMP treatment upregulated genes involved in membrane protein insertion (*yidC*, *yajC*) [45,46], sugar transport and polysaccharide synthesis (*agaB*, *agaC*), and membrane fatty acid synthesis (*RS02005*, *plsY*). These changes led to significant increases in extracellular protein, extracellular and membrane polysaccharides, and membrane fatty acids in G-Y12 (*p* < 0.05). Specifically, YidC and YajC facilitate membrane protein folding and insertion; the AgaB/AgaC PTS mediates sugar transport for polysaccharide synthesis; RS02005 is involved in lipoteichoic acid production; and PlsY functions as a glycerol-3-phosphate acyltransferase in fatty acid synthesis [47]. These modifications are consistent with the elevated membrane fatty acid levels (Table 1) and enhanced stress tolerance observed in G-Y12 (Figure 2C).

The L-lactate dehydrogenase encoded by RS11520 catalyzes the conversion of pyruvate to L-lactic acid, regenerating NAD^+^ to sustain glycolysis and enabling rapid energy production via substrate-level phosphorylation [48,49]. In contrast, the F-type ATPase subunits encoded by *atpB* and *atpF*, which typically synthesize ATP through oxidative phosphorylation, were downregulated, potentially reducing energy consumption. This differential expression—upregulation of glycolytic LDH and downregulation of ATP synthase—reflects a metabolic shift in G-Y12 that balances energy generation with stress tolerance.

## 5. Conclusions

In conclusion, this study demonstrated that the exogenous supplementation of GMP could promote the accumulation of intracellular second messengers C-di-AMP and C-di-GMP in Y12 cells, thereby enhancing the biofilm formation ability of Y12, promoting the synthesis of polysaccharides and protein substances, and also altering the composition of membrane fatty acids. These phenotypic results correspond to the expression levels of related enzyme genes in the transcriptome analysis. This research provides certain data support for probiotics to enhance their ability to withstand external environmental stress, and offers theoretical support for promoting the application and development of the probiotic industry.

## Figures and Tables

**Table 1:** Effect of GMP on membrane lipid fatty acid composition of Y12. "↑" and "↓" indicate that the fatty acid content in the G-Y12 group is higher or lower than that in the Y12 group.

Metabolite	Formula	Y12 (μg/mg)	G-Y12 (μg/mg)	Regulate	*p*-Value
palmitate	C_16_H_32_O_2_	0.033458 ± 0.001918	0.045335 ± 0.001315	↑	0.001
oleate	C_18_H_34_O_2_	0.006171 ± 0.001204	0.009616 ± 0.001312	↑	0.029
vaccenate	C_18_H_34_O_2_	0.005953 ± 0.001193	0.009281 ± 0.000840	↑	0.017
stearate	C_18_H_36_O_2_	0.005099 ± 0.001338	0.006790 ± 0.000789	↑	0.132
7-transnonadecenoate	C_19_H_36_O_2_	0.003451 ± 0.000527	0.004459 ± 0.000081	↑	0.031
myristate	C_14_H_28_O_2_	0.001789 ± 0.000265	0.002434 ± 0.000019	↑	0.014
palmitoleate	C_16_H_30_O_2_	0.001480 ± 0.000228	0.001870 ± 0.000223	↑	0.101
10-transnonadecenoate	C_19_H_36_O_2_	0.001448 ± 0.000049	0.001629 ± 0.000040	↑	0.008
trans 11-eicosenoate	C_20_H_38_O_2_	0.001247 ± 0.000182	0.001391 ± 0.000013	↑	0.244
arachidonate	C_20_H_32_O_2_	0.001183 ± 0.000345	0.001308 ± 0.000264	↑	0.645
linoleate	C_18_H_32_O_2_	0.000863 ± 0.000076	0.001341 ± 0.000206	↑	0.020
myristoleate	C_14_H_26_O_2_	0.000737 ± 0.000044	0.000732 ± 0.000170	↓	0.969
palmitelaidate	C_16_H_30_O_2_	0.000719 ± 0.000036	0.001055 ± 0.000087	↑	0.003
11-14 eicosadienoate	C_20_H_36_O_2_	0.000637 ± 0.000224	0.000595 ± 0.000166	↓	0.809
linoelaidate	C_18_H_32_O_2_	0.000605 ± 0.000013	0.000725 ± 0.000007	↑	0.001
11-14-17 eicosatrienoate	C_20_H_34_O_2_	0.000454 ± 0.000032	0.000460 ± 0.000039	↑	0.845
10-pentadecenoate	C_15_H_28_O_2_	0.000453 ± 0.000109	0.000469 ± 0.000071	↑	0.839
myristelaidate	C_14_H_26_O_2_	0.000317 ± 0.000084	0.000340 ± 0.000079	↑	0.749
petroselaidate	C_18_H_34_O_2_	0.000309 ± 0.000026	0.000438 ± 0.000035	↑	0.006
elaidate	C_18_H_34_O_2_	0.000187 ± 0.000031	0.000285 ± 0.000013	↑	0.007
pentadecanoate	C_15_H_30_O_2_	0.000096 ± 0.000041	0.000102 ± 0.000051	↑	0.887
heptadecanoate	C_17_H_34_O_2_	0.000077 ± 0.000019	0.000094 ± 0.000028	↑	0.448
arachidate	C_20_H_40_O_2_	0.000069 ± 0.000003	0.000083 ± 0.000007	↑	0.028
laurate	C_12_H_24_O_2_	0.000036 ± 0.000004	0.000051 ± 0.000005	↑	0.020
tridecanoate	C_13_H_26_O_2_	0.000018 ± 0.000002	0.000018 ± 0.000009	↑	0.904
caprate	C_10_H_20_O_2_	0.000012 ± 0.000001	0.000011 ± 0.000002	↓	0.758
caprylate	C_8_H_16_O_2_	0.000004 ± 0.000002	0.000004 ± 0.000003	↓	0.822

**Table 2:** Key differentially expressed genes of Y12 under the influence of GMP.

Pathway	Gene ID	Name	FC	Regulate	KEGG	Description
\	C7M33_RS13870	RS13870	1.62	UP	\	LuxR family transcriptional regulator
Quorum sensing (ko02024)	C7M33_RS12865	yidC	1.33	UP	K03217	membrane protein insertase YidC
	C7M33_RS01105	yajC	1.35	UP	K03210	preprotein translocase subunit YajC
	C7M33_RS05235	RS05235	1.30	UP	K01580	glutamate decarboxylase
Two-component system (ko02020)	C7M33_RS14760	dltA	1.26	UP	K03367	D-alanine--poly(phosphoribitol) ligase subunit DltA
	C7M33_RS09475	rpoN	1.43	UP	K03092	RNA polymerase factor sigma-54
Purine metabolism(ko00230)	C7M33_RS04640	RS04640	0.82	down	K01120	3′,5′-cyclic-nucleotide phosphodiesterase
Peptidoglycan biosynthesis (ko00550)	C7M33_RS09605	murB	1.27	UP	K00075	UDP-N-acetylmuramate dehydrogenase
Phosphotransferase system (PTS) (ko02060)	C7M33_RS02215	agaB	1.46	UP	K10984	PTS galactosamine transporter subunit IIB
	C7M33_RS02210	agaC	1.37	UP	K10985	PTS galactosamine transporter subunit IIC
Glycerolipid metabolism (ko00561)	C7M33_RS14075	plsY	1.28	UP	K08591	glycerol-3-phosphate 1-O-acyltransferase PlsY
	C7M33_RS02005	RS02005	1.31	UP	K19005	LTA synthase family protein
Glycolysis/Gluconeogenesis (ko00010)	C7M33_RS11520	RS11520	1.32	UP	K00016	L-lactate dehydrogenase
Oxidative phosphorylation (ko00190)	C7M33_RS01495	atpB	0.63	down	K02108	F0F1 ATP synthase subunit A
	C7M33_RS01485	atpF	0.90	down	K02109	F0F1 ATP synthase subunit B

## Data Availability

The original contributions presented in this study are included in the article. Further inquiries can be directed to the corresponding author.
